# Functional outcomes in oncology patients receiving chiropractic care: a retrospective chart review

**DOI:** 10.1007/s00520-026-11248-y

**Published:** 2026-09-23

**Authors:** Scott Siegel, Karina Szymulanska-Ramamurthy, Digant Gupta

**Affiliations:** 1 Integrative Medicine Department, City of Hope Atlanta, 600 Celebrate Life Parkway, Newnan, GA 30265 USA; 2 Department of Medical Oncology and Therapeutics Research, City of Hope Chicago, Chicago, IL USA

**Keywords:** Cancer, Oncology, Pain, Function, Chiropractic supportive care, Integrative medicine

## Abstract

**Purpose:**

Oncology patients often experience musculoskeletal (MSK) disorders because of common cancer treatments such as chemotherapy, radiation therapy, hormone-blocking medication, and surgery. These side effects include higher pain levels and a decreased range of motion, which can negatively impact activities of daily living. With a focus on neuromuscular health, chiropractic care, which uses low-force spinal manipulative therapy and myofascial techniques, may offer safe conservative treatment to help address these challenges.

**Methods:**

This single-arm retrospective observational study evaluated the relationship between chiropractic care and patient-specific functional scale (PSFS) and pain intensity in a sample of 97 oncology patients in a regional comprehensive cancer hospital setting. Using documented PSFS scores, the study analyzed pre- and post-treatment functional changes in activities and assessed their statistical significance.

**Results:**

Data analysis showed statistically significant improvements in patient-specific function and pain intensity across visits (*p* < 0.001). Linear mixed-effects models demonstrated a mean PSFS increase from 4.45 at the baseline visit to 5.13 at FU1 and 5.53 at FU2. Mean pain intensity decreased from 6.07 at the baseline visit to 5.10 at FU1 and 4.64 at FU2.

**Conclusion:**

These preliminary findings demonstrated improvements in patient-specific function and pain intensity across visits among oncology patients receiving chiropractic care. Although study limitations include the absence of a control group and variability in treatment techniques, improvements in specific function and pain intensity encourage further prospective controlled research to evaluate how chiropractic can be integrated into supportive oncology care.

## Introduction

Cancer treatments such as chemotherapy, radiation therapy, hormone therapy, and surgery are frequently associated with a high burden of musculoskeletal (MSK) disorders [1]. These side effects can impair physical function, exacerbate preexisting MSK conditions, and significantly reduce quality of life [2]. Cancer survivors face a threefold increased risk of functional impairment compared to the general population, often resulting in chronic disability [2]. Common consequences include pain, reduced range of motion, postural changes, and difficulty performing activities of daily living [3, 4]. Rehabilitation outcomes data also show a relationship between pain and poorer function in oncology survivor patients [2, 5]. Modern oncology aims to go beyond extending life to include integrative care efforts which address related quality of life challenges. At the same time, patients are generally utilizing various forms of integrative medicine more frequently [5]. The interdisciplinary nature of comprehensive cancer centers necessitates a wide range of services to manage and meet the multiple and interrelated needs of cancer patients. Cancer centers are increasingly incorporating supportive and integrative services to better address these challenges [6]. Supportive care in cancer focuses on the prevention and management of the adverse effects of cancer and its treatment across the entire cancer continuum, including physical effects [7]. Integrative medicine is defined by the World Health Organization as an interdisciplinary and evidence-based approach to health and well-being by using a combination of biomedical and traditional and/or complementary medical knowledge, skills, and practices [8].

Chiropractic care, particularly low-force and instrument-assisted techniques, offers a conservative, nonpharmacological approach to managing neuromusculoskeletal complications in oncology patients [2, 7]. By improving joint mobility, reducing muscle hypertonicity, and enhancing neurological function, chiropractic interventions may address conditions such as postsurgical adhesions, radiation-induced fibrosis, chemotherapy-induced peripheral neuropathy (CIPN), and hormone therapy-related arthralgias [1, 3, 7]. Despite its potential, there is a lack of published research evaluating the impact of spinal manipulation and chiropractic care on functional outcomes in this population [9]. A few osteopathic studies were identified, though which evaluated the use of manual therapy as a form of supportive oncology care to improve pain intensity and/or quality of life [10–13]. However, these studies also require further investigation.

This study is designed to evaluate the relationship between chiropractic care and functional outcomes using the Patient-Specific Functional Scale (PSFS), a validated tool for assessing individualized functional goals [14–17]. A literature search revealed no prior studies analyzing PSFS outcomes in oncology patients receiving chiropractic care, highlighting a critical gap in evidence [18]. The retrospective chart review study design allows for real-world assessment of functional changes in patients treated within an integrative oncology setting, providing foundational data for future controlled trials [16, 17]. This study aims to be the first preliminary study to quantify functional improvements in cancer patients receiving chiropractic care. Our hypothesis is that oncology patients receiving chiropractic care would demonstrate improvements in both specific function and pain intensity between baseline recording and during two subsequent follow-up visits.

## Methods

### Study design

This study employed a retrospective chart review using a single‑arm pre–post design without a concurrent control group.

### Setting

The study was conducted within the Integrative Medicine Department at City of Hope Cancer Center Atlanta a single-arm urban academic medical center in Newnan, Georgia.

### Participants

All adult patients aged 18–99 with a documented cancer diagnosis who received chiropractic care during the 12-month study period from June 1, 2024, to June 1, 2025, and had PSFS and pain intensity scores documented before treatment at the baseline visit and the next two eligible visits were included. Eligible patients had musculoskeletal complaints appropriate for chiropractic intervention and had undergone cancer treatment during the study window. Patients were excluded if required outcome measures were missing; if they received only one chiropractic visit or had no eligible follow-up within 3 months; if concurrent physical therapy, orthopedic surgery, massage therapy, acupuncture, pain medication, recent corticosteroid injection, or other interventions could confound outcomes; or if manual chiropractic care was contraindicated because of clinical complexity or safety concerns, including severe osteoporosis, extremely low platelet counts, acute compression fracture, recent surgery, vertebral artery stenosis, blood clots, or cauda equina syndrome [19]. Charts were also screened for indwelling devices, recent surgery, and osseous metastases identified on imaging.

### Recruitment procedures and oversight

Institutional Review Board approval was obtained before study procedures began. This retrospective chart review involved no direct patient contact. Eligible records were identified through electronic medical-record queries, and demographic information, diagnostic categories, visit dates, chiropractic treatment information, and functional and pain outcomes were extracted from the records.

### Chiropractic intervention

Chiropractic care was defined as individualized, modified neuromusculoskeletal care delivered by a licensed chiropractor in an oncology clinic. Interventions included low-force or instrument-assisted spinal or extremity manipulation, myofascial techniques, exercise instruction, ergonomic advice, activity of daily living modification, and patient education. High-velocity, low-amplitude spinal manipulation was not used because of potential oncology-related structural vulnerabilities.

### Outcomes

The Patient-Specific Functional Scale (PSFS) assesses activities identified by each patient as difficult to perform. In this study, one patient-selected activity/function was tracked per patient across the three ordered visits. The activity was scored from 0 (unable to perform) to 10 (able to perform at the prior level), with higher scores indicating better function [14–17]. Pain intensity was recorded on an 11-point numerical rating scale ranging from 0 (no pain) to 10 (worst pain), and lower scores are more favorable [22, 23]. Both measures have documented reliability, validity, and responsiveness in musculoskeletal and rehabilitation populations [15–17, 22, 23]. Outcomes were recorded before treatment at three ordered visits: the baseline visit, the first eligible subsequent visit (FU1), and the second eligible subsequent visit (FU2). FU1 and FU2 therefore indicate visit order rather than standardized elapsed-time points.

### Data analysis

Descriptive statistics summarized patient characteristics, outcome measures, and intervals between visits. Continuous variables were reported as means with standard deviations and categorical variables as counts and percentages. FU1 assessment before care reflected potential effects of treatment delivered at the baseline visit, together with the usual confounding influences inherent in this retrospective design. FU2 assessment before care reflected potential combined effects of treatment delivered at the baseline and FU1 visits. Because clinical scheduling was not protocolized, visit order did not correspond to fixed elapsed-time points.

Changes in PSFS and pain intensity across visits were evaluated using linear mixed-effects models to account for within-subject correlation across repeated measurements [20, 21]. For each outcome, visit (baseline, FU1, and FU2) was included as a categorical fixed effect and subject as a random intercept. The overall effect of visit was reported. Pairwise comparisons were conducted for baseline versus FU1, baseline versus FU2, and FU1 versus FU2; mean differences (I−J), standard errors, 95% confidence intervals, and Holm–Bonferroni-adjusted *p*-values were reported.

Clinically meaningful change was additionally evaluated using responder analyses. Patients were considered responders if their change in score met or exceeded predetermined thresholds. For PSFS, a minimal clinically important difference (MCID) was defined as an improvement of ≥ 2.0 points from baseline, while for pain intensity a clinically meaningful response was defined as a ≥ 2.0-point decrease from baseline [22, 23]. The proportion of responders at FU1 and FU2 was calculated for each outcome, with 95% confidence intervals estimated using the Wilson method, to characterize the proportion of patients achieving clinically important improvement across visits.

To examine whether outcome patterns across visits differed by patient characteristics, the LMMs were extended to include between-subject factors and their interactions with visit for PSFS and pain intensity. Separate models were fitted to evaluate visit × gender (male vs female) and visit × race (White vs African American) interactions. Statistical significance of the interaction terms was used to determine whether changes across visits differed among groups. When interactions were not significant, results were interpreted as indicating comparable patterns across visits among groups. Model-based mean patterns were visualized using line plots displaying group-specific means at the baseline, FU1, and FU2 visits.

All statistical tests were two-sided, and a *p*-value < 0.05 was considered statistically significant unless otherwise specified. Analyses appropriately accounted for within-subject correlation across repeated visit measurements. All statistical analyses were performed using IBM SPSS Statistics, v29. Graphical displays accompanied numerical results to aid interpretation of patterns across visits.

## Results

A total of 97 adult oncology patients met the eligibility criteria and had the required outcome data. Patient demographics and oncology diagnosis categories are summarized in the patient-characteristics table.

The cohort had a mean age of 57.4 years and was predominantly female. White and African American patients comprised the largest racial groups, and breast cancer was the most common oncology diagnosis category (Table 1). Patients received low-force instrument-assisted spinal and extremity manipulation, myofascial techniques, flexion-distraction, therapeutic exercise instruction, and ergonomic or activity counseling.

**Table 1:** Patient demographic and oncology characteristics (*N* = 97)

Characteristic	Value
**Age, years**	
Mean ± SD	57.4 ± 12.1
Range	27–83
**Gender, *n* (%)**	
Women	78 (80.4)
Men	19 (19.6)
**Race, *n* (%)**	
White	49 (50.5)
African American	38 (39.2)
Other	5 (5.2)
Not recorded	5 (5.2)
**Oncology diagnosis category, *n* (%)**	
Breast	55 (56.7)
Gastrointestinal	12 (12.4)
Genitourinary	9 (9.3)
Gynecological	7 (7.2)
Hematological	6 (6.2)
Head and neck	3 (3.1)
Thyroid	3 (3.1)
Skin	2 (2.0)

The timing of follow-up visits varied across patients. The mean interval from the baseline visit to FU1 was 18.3 ± 19.2 days, with a median of 14 days (IQR, 7–23; range, 1–130). The mean interval from the baseline visit to FU2 was 37.5 ± 32.3 days, with a median of 26 days (IQR, 14–54; range, 2–184). The mean interval from FU1 to FU2 was 19.3 ± 20.0 days, with a median of 14 days (IQR, 7–21; range, 1–136) (Tables 2 and 3).

**Table 2:** Timing of baseline, FU1, and FU2 scores

Interval	Mean ± SD (days)	Median (IQR), days	Range, days
Baseline to FU1	18.3 ± 19.2	14 (7–23)	1–130
Baseline to FU2	37.5 ± 32.3	26 (14–54)	2–184
FU1 to FU2	19.3 ± 20.0	14 (7–21)	1–136

**Table 3:** Repeated measures for PSFS and pain intensity across three visits

Outcome	Baseline visit, mean ± SD	FU1 visit, mean ± SD	FU2 visit, mean ± SD	Overall visit *p*-value
PSFS	4.45 ± 1.42	5.13 ± 1.36	5.53 ± 1.43	< 0.001
Pain intensity	6.07 ± 1.60	5.10 ± 1.48	4.64 ± 1.49	< 0.001

Values are mean ± SD at the baseline, first follow-up (FU1), and second follow-up (FU2) visits. FU1 and FU2 indicate visit order rather than standardized elapsed-time points. Overall *p*-values are from the linear mixed-effects models. Two-sided *α* = 0.05

Pairwise comparisons confirmed statistically significant improvement between each ordered visit for both outcomes (Table 4). PSFS scores increased from baseline to FU1 (mean difference [baseline − FU1], −0.680; 95% CI, −0.854 to −0.507; adjusted *p* < 0.001), from baseline to FU2 (−1.077; 95% CI, −1.329 to −0.826; adjusted *p* < 0.001), and from FU1 to FU2 (−0.397; 95% CI, −0.588 to − 0.206; adjusted *p* < 0.001).

**Table 4:** Pairwise comparisons of PSFS and pain intensity across visits

Outcome	Comparison (I−J)	Mean difference	SE	95% CI	Adjusted *p*-value
**PSFS**	Baseline vs FU1	− 0.680	0.087	− 0.854 to − 0.507	< 0.001
	Baseline vs FU2	− 1.077	0.127	− 1.329 to − 0.826	< 0.001
	FU1 vs FU2	− 0.397	0.096	− 0.588 to − 0.206	< 0.001
**Pain intensity**	Baseline vs FU1	0.933	0.102	0.730 to 1.136	< 0.001
	Baseline vs FU2	1.423	0.143	1.139 to 1.706	< 0.001
	FU1 vs FU2	0.490	0.120	0.252 to 0.727	< 0.001

Mean differences are expressed as I−J. Negative PSFS differences indicate higher function at the later visit; positive pain intensity differences indicate lower pain at the later visit. *p*-values are Holm–Bonferroni adjusted

Pain intensity decreased from baseline to FU1 (mean difference [baseline − FU1], 0.933; 95% CI, 0.730 to 1.136; adjusted *p* < 0.001), from baseline to FU2 (1.423; 95% CI, 1.139 to 1.706; adjusted *p* < 0.001), and from FU1 to FU2 (0.490; 95% CI, 0.252 to 0.727; adjusted *p* < 0.001).

Clinically meaningful improvement, defined as a ≥ 2-point increase in PSFS or a ≥ 2-point decrease in pain intensity from baseline, occurred in 13.4% and 22.7% of patients, respectively, at FU1 and in 26.8% and 44.3%, respectively, at FU2.

Functional status improved across visits in both males and females. Among males, mean PSFS increased from the baseline visit through FU1 and FU2, and a similar pattern was observed among females, with higher PSFS scores at each successive visit compared with baseline. Although females had slightly higher PSFS scores than males at all three visits, the overall pattern and magnitude of improvement were comparable by gender. Consistent with this observation, the visit × gender interaction was not statistically significant (*p* = 0.986), indicating that sex did not modify the pattern of functional improvement across visits. These findings suggest that chiropractic care was associated with similar functional gains in male and female patients across the observed visits. FU1 and FU2 indicate visit order and do not represent standardized elapsed-time points because follow-up scheduling varied among patients. The figure is descriptive and should not be interpreted as demonstrating a uniform elapsed-time trajectory.

Both White and African American patients demonstrated improvements in PSFS scores from the baseline visit to FU1 and FU2. Mean PSFS values increased at each successive visit in both racial groups, with no evidence of divergence in the pattern or magnitude of improvement across visits. Consistent with this observation, the visit × race interaction was not significant (*p* = 0.325), suggesting that race did not modify the functional response across visits. Overall, chiropractic care was associated with similar functional gains among White and African American patients. Because assessment timing varied among patients, the figure is intended to display descriptive mean patterns by visit order only.

Pain intensity decreased across visits in both males and females. Among males, mean pain intensity scores declined from the baseline visit through FU1 and FU2, and a similar pattern was observed among females, with lower scores at each successive visit compared with baseline. Although females reported slightly higher pain intensity scores than males at all three visits, the overall pattern and magnitude of reduction were comparable by gender. Consistent with this observation, the visit × gender interaction was not statistically significant (*p* = 0.440), indicating that gender did not modify the pattern of pain intensity reduction across visits. These findings suggest that chiropractic care was associated with similar reductions in pain intensity among male and female patients across the observed visits. The figure is descriptive and should not be interpreted as demonstrating a standardized elapsed-time pattern of pain intensity change.

**Fig. 1:**
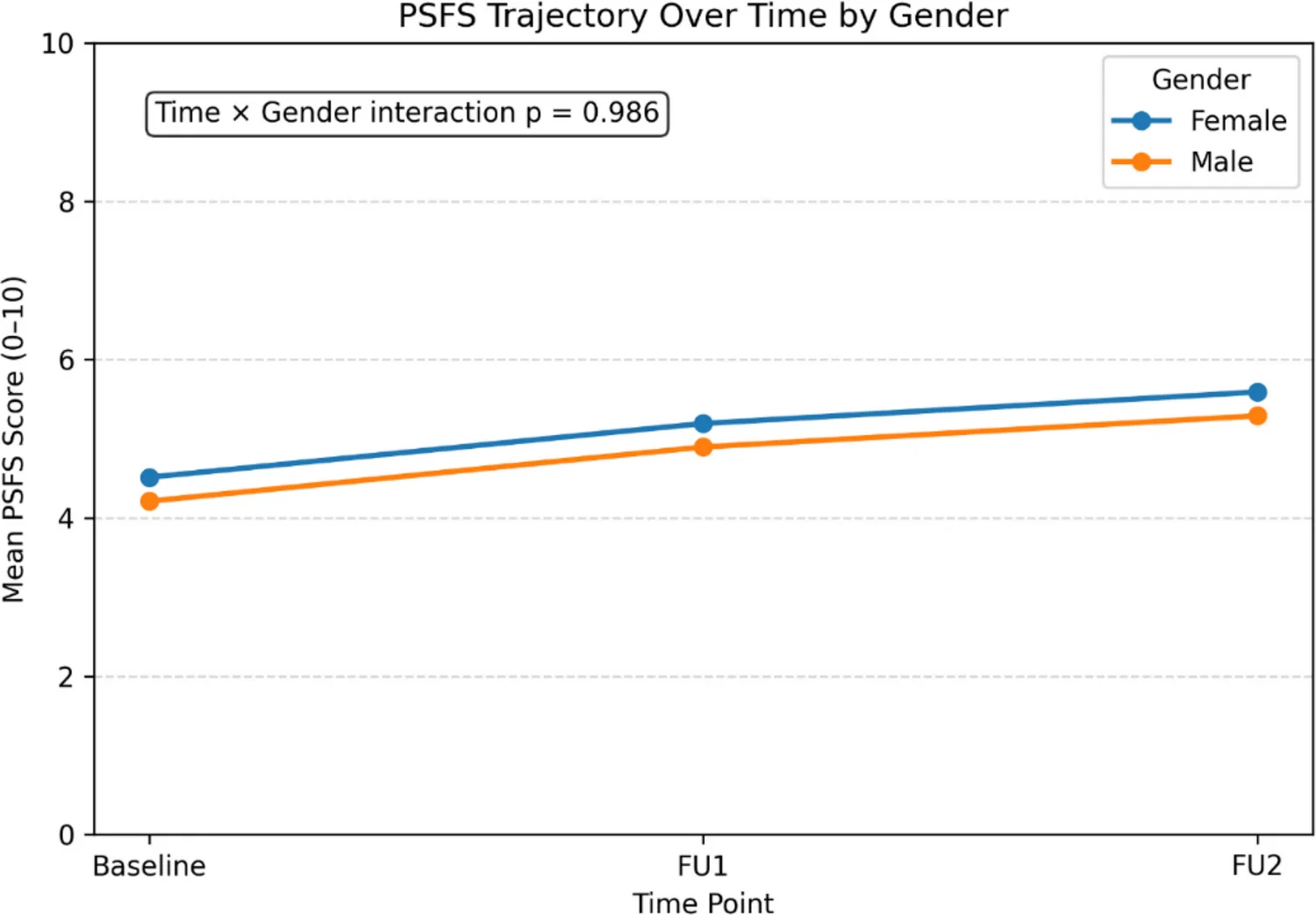
PSFS trajectory by gender over visits

**Fig. 2:**
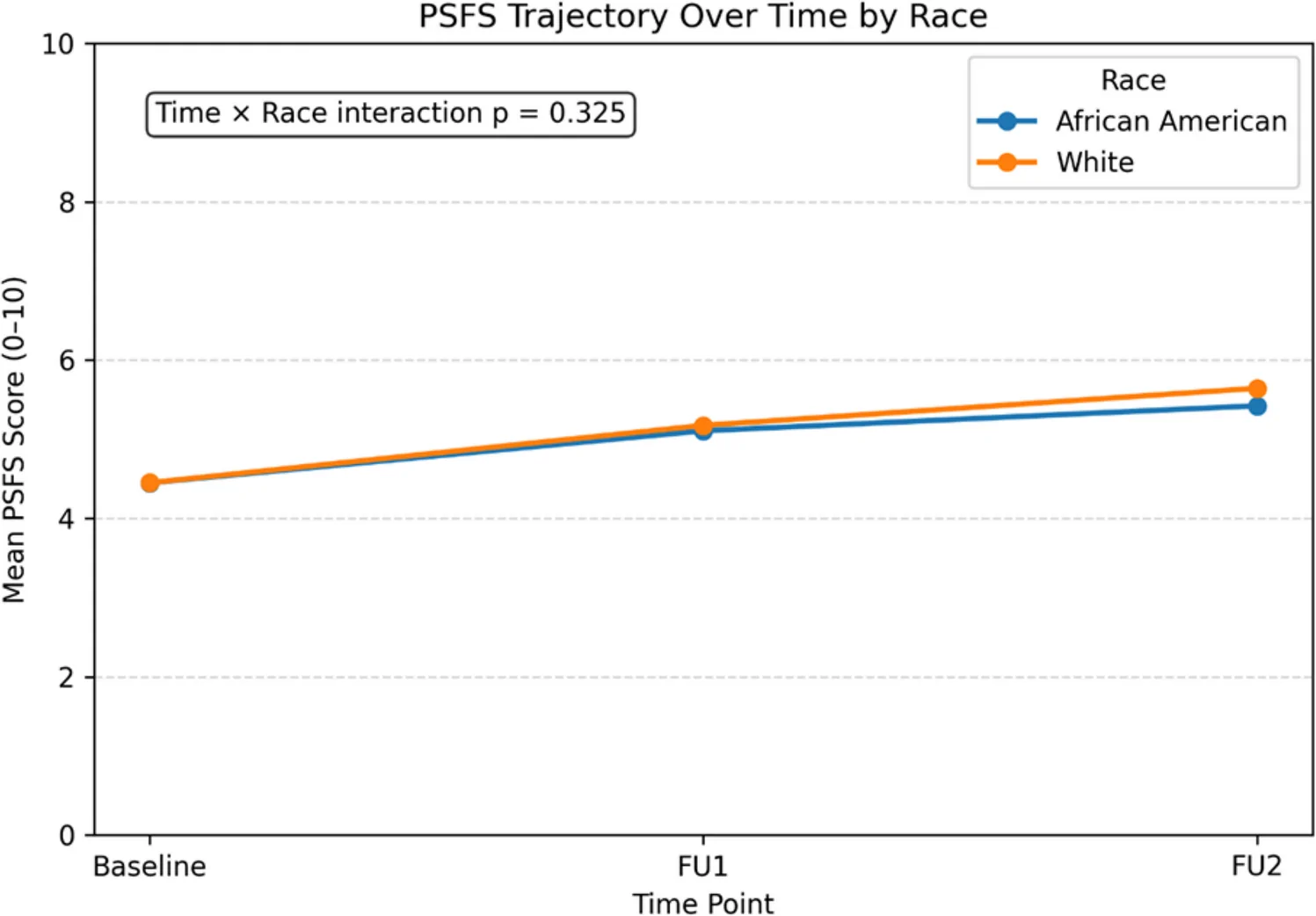
PSFS trajectory by race over visits

**Fig. 3:**
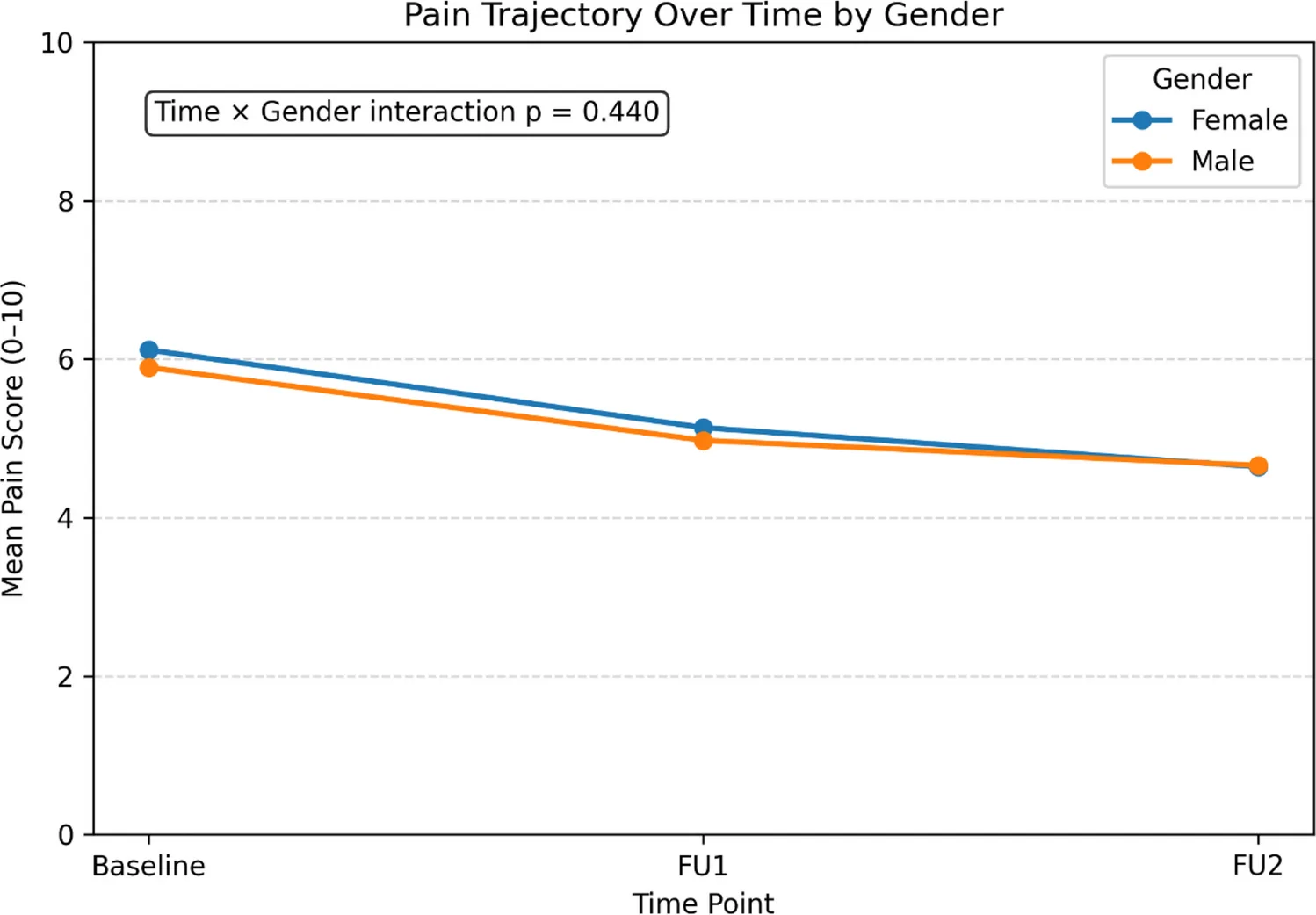
Pain trajectory by gender over visits

**Fig. 4:**
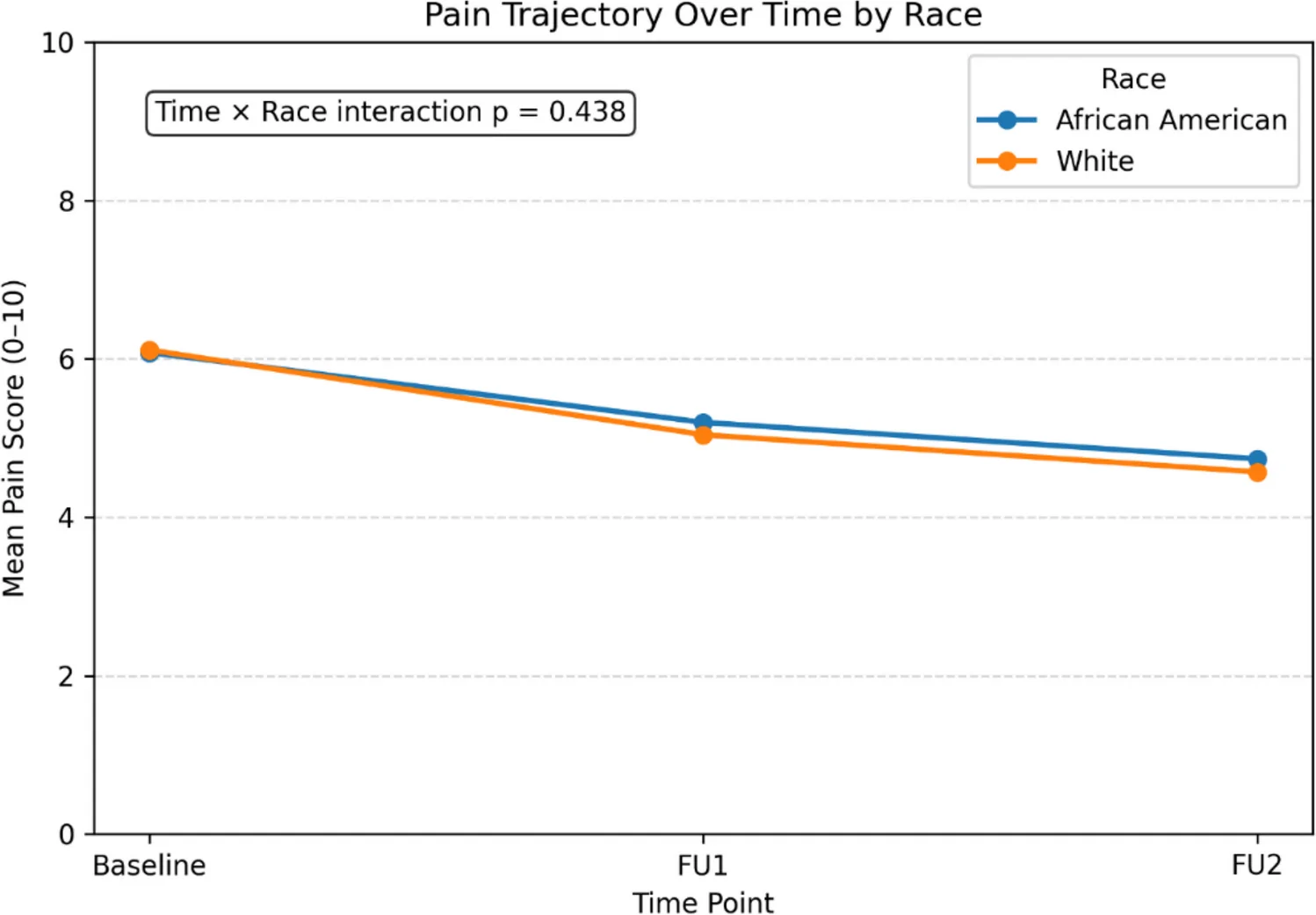
Pain intensity trajectory by race over visits

Both White and African American patients demonstrated reductions in pain intensity scores from the baseline visit to FU1 and FU2. Mean pain intensity values decreased at each successive visit in both racial groups, with no evidence of divergence in the pattern or magnitude of reduction across visits. Consistent with this observation, the visit × race interaction was not significant (*p* = 0.438), suggesting that race did not modify the pain intensity response across visits. Overall, chiropractic care was associated with similar reductions in pain intensity among White and African American patients. Figures 1, 2, 3, and 4 present descriptive mean values by visit order. These plots should not be interpreted as depicting standardized elapsed-time trajectories.

## Discussion

In this retrospective analysis of oncology patients receiving chiropractic care, both functional status and pain intensity demonstrated statistically significant improvements across visits, with consistent increases in PSFS scores and reductions in pain intensity scores from the baseline visit through FU1 and FU2. Clinically meaningful improvements were observed in a subset of patients, with the proportion of responders increasing at successive visits for both function and pain intensity. Subgroup analyses further showed that these visit-based patterns were consistent across sex and race, with no evidence of differential response. The consistency of findings across demographic subgroups supports the robustness of the observed patterns.

From a clinical perspective, these findings suggest that chiropractic care may offer a useful nonpharmacological option for managing MSK symptoms in oncology patients. Improvements in function and reductions in pain intensity, even when modest at the group level, may translate into meaningful benefits in daily activities, mobility, and overall quality of life for patients undergoing or recovering from cancer treatment. Given the growing emphasis on supportive and integrative care in oncology, chiropractic interventions may represent a complementary approach that can be incorporated alongside standard medical management to address symptom burden. However, careful patient selection and coordination with the oncology care team remain important to ensure safety and appropriateness of care [24].

This study has several limitations. First, the retrospective, single-arm pre–post design without a concurrent control group precludes causal inference; observed changes may reflect natural recovery, regression to the mean, or concurrent oncologic or supportive treatments. Analgesic medication, rehabilitation therapy, and palliative care treatment could not be fully controlled during the observation period. Second, the single-center sample was modest, limiting subgroup power and generalizability. Third, self-reported outcomes are subject to reporting bias and may be influenced by expectations or recall. Fourth, follow-up timing definitely varied across patients, producing heterogeneous intervals and treatment exposure; FU1 and FU2 denote visit order, not common elapsed-time points. Finally, exploratory longitudinal and subgroup comparisons may be vulnerable to chance findings despite multiple testing adjustments.

Despite these limitations, the study has several strengths. It utilized repeated, patient-centered outcome measures, including both functional status and pain intensity, allowing for a more comprehensive assessment of patient-reported outcomes across visits. The evaluation of both statistically significant changes and clinically meaningful improvements enhances the interpretability and clinical relevance of the findings. The availability of two follow-up visits enabled assessment of patterns by visit order rather than reliance on a single post-intervention measurement. In addition, the inclusion of subgroup analyses by sex and race provides insights into the consistency of effects across key patient characteristics. Finally, the use of repeated-measures analytical approaches that account for within-subject correlation strengthens the robustness of the findings.

The next research step should be a properly powered prospective randomized efficacy or effectiveness trial with an appropriate comparison group. Such a study should use standardized assessment windows, document co-interventions, and evaluate the durability of changes in function and pain intensity. Subsequent multicenter studies may then be appropriate to assess generalizability across oncology settings and cancer types. Mixed-methods and pragmatic research could further clarify patient experience, optimal treatment frequency and components, and implementation within comprehensive cancer care pathways.

## Conclusion

In conclusion, receipt of chiropractic care among diverse adult oncology patients was associated with improvements in functional status and reductions in pain intensity across visits, as reflected by changes in mean scores across the cohort and clinically meaningful improvements observed in some patients. Functional and pain intensity outcomes improved from the baseline visit through FU1 and FU2, with no evidence that these visit-based patterns differed by gender or race. Given the observational, single-arm design, these findings should be interpreted cautiously, as causal inferences cannot be made. Nevertheless, the observed patterns suggest that chiropractic care may have a potential supportive role in oncology care, warranting further evaluation in controlled, prospective studies to better establish effectiveness and inform clinical integration.

## Data Availability

The datasets generated during and/or analyzed during the current study are available from the corresponding author on reasonable request.
